# How Sociodemographic, Water, and Sanitation Factors Influence Diarrhea in Children Under Five: Insights from Indonesia’s Underdeveloped Regions

**DOI:** 10.34172/jrhs.2025.171

**Published:** 2024-12-25

**Authors:** Sailent Rizki Sari Simaremare, Basuki Rachmat, Wahyu Pudji Nugraheni, Debri Rizki Faisal, Muhammad Nirwan, Mara Ipa, Tities Puspita, Dea Anita Ariani Kurniasih, Felly Philipus Senewe

**Affiliations:** ^1^Research Center for Public Health and Nutrition, Health Research Organization, National Research and Innovation Agency, Cibinong Bogor, West Java, Indonesia

**Keywords:** Diarrhea, Child, Risk factors, Rural population, Indonesia

## Abstract

**Background:** Despite the decrease in prevalence from 18.5% in 2013 to 12.3% in 2018, diarrhea presents a major public health challenge in Indonesia which leads to significant mortality. This study investigated factors influencing diarrhea among children under five years of age in underdeveloped regions of Indonesia, where disparities from other regions are significant.

**Study Design:** A cross-sectional study.

**Methods:** This study obtained data from National Basic Health Research conducted in 2018. Sixty underdeveloped regions of Indonesia, with a total of 9243 children aged 0-59 months, were included. Chi-square, bivariate, and multivariate analyses were conducted to determine factors influencing the prevalence of diarrhea in children under 5 years of age.

**Results:** Multivariate analysis revealed that the age categories of 12-23 months (OR: 1.73; 95% CI: 1.48, 2.02) and 24-35 months (OR: 1.31; 95% CI: 1.11, 1.53), awareness of a nearby hospital (OR: 0.74; 95% CI: 0.63, 0.86), and history of acute respiratory infection (ARI) in the past month (OR: 1.99; 95% CI: 1.66, 2.40) were associated with diarrhea in children under the age of five in underdeveloped regions of Indonesia. In contrast, the environmental factors analyzed further in the study were not significantly associated with the prevalence of diarrhea in children under five years of age in underdeveloped regions of Indonesia.

**Conclusion:** These findings suggest that the child’s age, the child’s history of ARI, and household awareness of nearby hospitals are critical factors associated with the child’s diarrhea in underdeveloped regions of Indonesia.

## Background

 Diarrhea poses a significant threat to public health not only in Indonesia but also globally, resulting in substantial mortality. In 2016, over 1.6 million deaths worldwide were attributed to the disease, with 26.93% of these fatalities occurring among children under the age of 5. Moreover, approximately 90% of deaths were concentrated in South Asia and sub-Saharan Africa.^1^ Diarrhea continues to be a leading cause of mortality among children under the age of five in Indonesia despite the decrease in prevalence from 18.5% in 2013 to 12.3% in 2018.^2^ Even though diarrhea is the leading cause of mortality among children under the age of five in South Asia, diarrhea-related deaths are largely preventable.^3^ Diarrhea is often linked to environmental factors such as drinking water quality and basic sanitation conditions. In a study conducted in Cameroon, diarrhea had a prevalence of 14.4%. Based on the results, the prevalence of diarrhea was associated with water supply and quality of drinking water; however, the findings varied among the neighborhoods.^4^ Another study from Kenya found that contaminated water sources were associated with the prevalence of waterborne diseases such as typhoid and diarrhea.^5^ Water and food contamination from open defecation practices in sub-Saharan Africa also potentially contributed to the occurrence of diarrhea.^6^ The combination of poor sanitation and unhealthy behavior resulted in a higher prevalence of diarrhea.^7^ These findings underscore the critical role of improving water quality, sanitation, and hygiene practices in mitigating the burden of diarrheal diseases, particularly in vulnerable regions.

 While numerous studies have been done to explore water and sanitation factors related to diarrhea, studies focusing on underdeveloped regions of Indonesia are still limited. Indonesia has many regions that remain remote and underdeveloped.^8^ Underdeveloped regions of Indonesia are typically poverty-stricken areas where access to healthcare and improved sanitation facilities are limited.^8,9^ Significant disparities in access to improved drinking water and sanitation facilities were found across different subnational regions, especially the underdeveloped regions.^10^ Addressing these disparities is crucial for reducing the prevalence of diarrhea and improving public health outcomes in most vulnerable communities in Indonesia. The prevalence of diarrhea in Indonesia exhibits significant regional disparities, with certain provinces like Papua, South Sulawesi, Aceh, West Sulawesi, and Central Sulawesi experiencing higher incidence and prevalence rates compared to other regions.^11^ Other studies suggested that the prevalence of diarrhea is exacerbated in the region with limited access to healthcare, safe water, and sanitation as well as low-income and marginalized communities.^12,13^ Therefore, we conducted this study to assess the prevalence of diarrhea and examine the influence of sociodemographic, water, and sanitation conditions on diarrhea in children under the age of five in underdeveloped regions of Indonesia. We hypothesized that sociodemographic factors, inadequate access to clean water, and poor sanitation play significant roles in diarrhea prevalence among children under the age of five in underdeveloped regions of Indonesia.

## Materials and Methods

###  Research design, participants, and data source 

 Data were obtained from National Basic Health Research (RISKESDAS) conducted in 2018. It was an observational cross-sectional study with multi-stage sampling based on provincial estimates. The study population comprised all households in Indonesia, with 30000 census blocks (CBs) selected using probability proportional to size (PPS) sampling method in each urban and rural district. From each CB, ten households were chosen, stratified by the highest education level of the head of household. In total, 295­720 households and 1­091­528 individuals were surveyed across 34 provinces. Children under five in districts classified as underdeveloped were included in the study. Based on the Indonesian Presidential Decree, underdeveloped regions are districts whose regions and people are less developed compared to other regions on a national scale based on criteria such as community economy, human resources, facilities and infrastructure, regional financial capacity, accessibility, and regional characteristics^14^. A total of 9243 children under five years of age were included in the study from 60 underdeveloped regions across the country.

###  Research variables

 The dependent variable in this study was diarrhea, determined by whether participants reported having been diagnosed with diarrhea within the past month by medical personnel. There were 14 independent variables in this study that covered sociodemographic, water, and sanitation information. Sociodemographic variables consisted of child’s gender, the child’s age which was classified into five categories such as 0-11 months, 12-23 months, 24-35 months, 36-47 months, and 48-59 months, and the child’s residential area which was classified as “rural” and “urban” areas. Considering the situation in underdeveloped regions of Indonesia, health facilities are still limited and often geographically distant from one another. The existence of health facilities was assessed based on whether the household was “aware of a nearby hospital” and “aware of a nearby public health center”, and the child’s medical history was determined by whether they “have been diagnosed with pneumonia within the past year” and “acute respiratory infection (ARI) within the past month”. Water and sanitation variables included clean water and drinking water sources, wastewater disposal from water closet, waste disposal from the kitchen, trash bins, household waste management, and child’s stool disposal.

 Clean water and drinking water sources were categorized into “improved” (packaged and delivered water, pipe water, boreholes, protected dug well, protected spring, and rainwater), “unimproved” (unprotected dug well and unprotected spring), and “surface water” (river, dam, lake, canal, or irrigation). Water closet and kitchen waste disposal were categorized into “improved” (with closed storage), “unimproved” (with open storage), and “open disposal” (without waste storage, disposed to open bodies of water). Household waste management was categorized into “properly” (carried away to garbage dump or buried), “composted”, “burned” and “at random” (on the ground, the ditch, and river). The child’s stool disposal was classified as “properly” (in the latrine) and “improperly” (buried, in the drain, ditch, or left in the open).

###  Statistical analysis

 Statistical analysis was performed with SPSS version 27.0. Factors associated with diarrhea in children under five years of age were analyzed using bivariate and multivariate logistic regression models. Chi-square analysis was applied in the initial bivariate analysis to assess whether differences in proportions across variable categories were statistically significant. The measures of association were expressed as odds ratios (ORs) with their corresponding 95% confidence intervals (CIs) for categorical variables. The geographical distribution of diarrhea prevalence in underdeveloped regions of Indonesia was obtained using QGIS version 3.36.2.

## Results

###  Sociodemographic and environmental characteristics

 The child’s age, ranging from 0-59 months, was classified into five age categories. Based on the results, 21.5% of the children were at the age of 24-35 months. Half of the children were male and most of them were living in rural areas (87.8%). Most of the children’s households were aware of a nearby hospital and public health center, with percentages of 78.8% and 97.5%, respectively. About 2.1% of the children were diagnosed with pneumonia in the past year and 9.7% of them were diagnosed with ARI in the past month. Improved drinking water and clean water sources were common (69.6% and 62.3%, respectively). Household waste management was poor, with uncovered trash bins (94.2%), improper stool disposal (62.8%), and open wastewater and kitchen waste disposal (84.3% and 86.4%, respectively).

###  Diarrhea prevalence in underdeveloped regions of Indonesia

**Figure 1:**
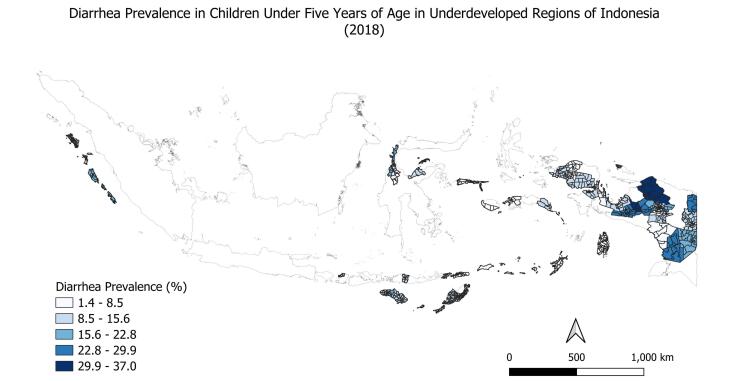
The geographical distribution of diarrhea prevalence in children under 5 years of age in underdeveloped regions of Indonesia

 The prevalence of diarrhea among children under the age of five in underdeveloped regions of Indonesia was 11.4%. Figure 1 illustrates the geographical distribution of the children included in the study. The prevalence of diarrhea varied across regions, with the highest rate being 31.8% (data not shown). Table 1 shows that the contribution of age categories to diarrhea prevalence was significantly different. Children aged 12-23 months had the highest prevalence of diarrhea (16.6%), followed by children aged 24-35 months (12.4%). No difference was observed in the prevalence of diarrhea between males (11.0%) and females (11.9%). Children living in rural areas (11.8%) had significantly a higher prevalence of diarrhea than those living in urban areas (8.9%). Diarrhea prevalence was significantly higher among children from households that were not “aware of a nearby hospital” (14.6%) compared to those that were “aware of a nearby hospital” (10.6%). However, it was not significantly higher in households that were “aware of a nearby public health center” (11.5%) than in those who were not “aware of a nearby public health center” (8.3%). Children under five years of age with a history of pneumonia (17.1%) and ARI (19.1%) had a significantly higher prevalence than those without infection. It was significantly higher in households with drinking water sources (13.8%) and clean water sources (16.1%) from surface waters. It was also significantly higher in households with improper child’s stool disposal (12.2%) and inadequate household waste management (12.7%). However, it was not significantly higher in households with uncovered trash bins (11.6%), open wastewater disposal (11.6%), and open kitchen waste disposal (11.6%).

**Table 1:** Proportion of diarrhea based on study variables among children under five years of age in underdeveloped regions of Indonesia (2018)

**Variables**	**Total**	**Diarrhea**				***P *** **value**
		**Yes**		**No**		
		**Number**	**Percent**	**Number**	**Percent**	
		**Socio-demographic**				
Age category (months)						0.001
1-11	1632	152	9.3	1480	90.7	
12-23	1781	278	16.6	1503	83.4	
24-35	1988	247	12.4	1742	87.6	
36-47	1866	198	10.6	1668	89.4	
48-59	1975	183	10.8	1792	89.2	
Gender						0.191
Male	4639	511	11.0	4128	89.0	
Female	4604	547	11.9	4057	88.1	
Residential area						0.003
Urban	1130	100	8.9	1030	91.1	
Rural	8113	958	11.8	7155	88.2	
Aware of a nearby hospital						0.001
Yes	7285	772	10.6	6513	89.4	
No	1958	286	14.6	1672	85.4	
Aware of a nearby public health center						0.130
Yes	9014	1039	11.5	7975	89.5	
No	229	19	8.3	210	91.7	
		**Medical history**				
Diagnosed with pneumonia in the past year						0.014
Yes	194	33	17.0	161	83.0	
No	9049	1024	11.3	8024	88.7	
Diagnosed with ARI in the past month						0.001
Yes	896	171	19.1	725	80.9	
No	8347	886	10.6	7461	89.4	
		**Water and sanitation**				
Drinking water source						0.053
Improved	6430	725	11.3	5705	88.7	
Unimproved	1902	207	10.9	1695	89.1	
Surface water	911	126	13.8	785	86.2	
Clean water source						0.005
Improved	5756	627	10.9	5129	89.1	
Unimproved	1978	221	12.6	1757	87.4	
Surface water	1509	209	16.1	1300	83.9	
Child’s stool disposal						0.005
Properly	3441	352	10.2	3090	89.8	
Improperly	5802	706	12.2	5095	87.8	
Trash bin with cover						0.148
Yes	536	51	9.5	485	90.5	
No	8707	1007	11.6	7701	88.4	
Household waste						0.022
Disposed properly	935	107	11.4	828	88.6	
Composted	96	8	8.3	88	91.7	
Burned	4731	502	10.6	4228	89.4	
At random	3481	442	12.7	3039	87.3	
Wastewater disposal						0.269
Improved	533	50	9.4	483	90.6	
Unimproved	921	102	11.1	819	88.9	
Open disposal	7789	906	11.6	6884	88.4	
Kitchen waste disposal						0.158
Improved	350	29	8.3	321	91.7	
Unimproved	906	102	11.3	805	88.7	
Open disposal	7987	927	11.6	7060	88.4	

###  Prevalence of diarrhea and related factors in underdeveloped regions of Indonesia

 Binary logistic regression analysis (Table 2) showed that diarrhea prevalence in children was significantly associated with the age categories of 11-23 months [OR: 1.80; 95% CI: 1.46, 2.22] and 24-35 months [OR: 1.38; 95% CI: 1.11, 1.71]. Children living in rural areas were more likely to suffer diarrhea than children living in urban areas [OR: 1.38; 95% CI: 1.11, 1.71]. The “Awareness of a nearby hospital” was associated with a decreased likelihood of diarrhea in children [OR: 0.69; 95% CI: 0.60, 0.80]. Other infectious diseases such as pneumonia [OR: 1.63; 95% CI: 1.11, 2.37] and ARI [OR: 1.99; 95% CI: 1.66, 2.38] were associated with higher diarrhea prevalence in children. In addition, drinking water sources [OR: 1.03; 95% CI: 1.03, 1.55] and clean water sources from surface waters [OR: 1.32; 95% CI: 1.11, 1.56], and household waste disposed of randomly [OR: 1.23; 95% CI: 1.07, 1.41] were significantly associated with higher diarrhea prevalence in children.

**Table 2:** Binary logistic regression analysis of study variables among children under five years of age in underdeveloped regions of Indonesia (2018)

**Variables**	**OR (95% CI)**	***P *** **value**
Female	1.09 (0.96, 0.24)	0.200
Age category (months)		
1-11	Ref.	
12-23	1.80 (1.46, 2.22)	0.001
24-35	1.38 (1.11, 1.71)	0.030
36-47	1.16 (0.93, 1.45)	0.201
48-59	0.99 (0.79, 1.25)	0.962
Living in rural area	1.38 (1.11, 1.71)	0.004
Aware of a nearby hospital	0.69 (0.60, 0.80)	0.001
Aware of a nearby public health center	1.44 (0.90, 2.32)	0.130
Diagnosed with ARI in the past month	1.99 (1.66, 2.38)	0.001
Diagnosed with pneumonia in the past year	1.63 (1.11, 2.37)	0.012
Drinking water source		
Improved	Ref.	
Unimproved	0.96 (0.82, 1.13)	0.622
Surface water	1.03 (1.03, 1.55)	0.026
Clean water source		
Improved	Ref.	
Unimproved	1.03 (0.88, 1.21)	0.709
Surface water	1.32 (1.12, 1.56)	0.001
Child’s stool disposed improperly	1.22 (1.06, 1.39)	0.004
Trash bin without cover	1.24 (0.92, 1.67)	0.156
Household waste		
Burned	Ref.	
Disposed properly	1.08 (0.87, 1.35)	0.477
Composted	0.72 (0.34, 1.52)	0.385
At random	1.23 (1.07, 1.41)	0.003
Wastewater disposal		
Improved	Ref.	
Unimproved	1.19 (0.83, 1.70)	0.338
Open disposal	1.26 (0.93, 1.70)	0.130
Kitchen waste disposal		
Improved	Ref.	
Unimproved	1.39 (0.91, 2.15)	0.131
Open disposal	1.45 (0.98, 2.13)	0.061

 Based on the results of multivariate logistic regression analysis (Table 3), the association between the prevalence of diarrhea and age categories of 12-23 months [OR: 1.73; 95% CI: 1.48, 2.02] and 24-35 months [OR: 1.31; 95% CI: 1.11, 1.53], awareness of a nearby hospital [OR: 0.74; 95% CI: 0.63, 0.86], and history of ARI in the past month [OR: 1.99; 95% CI: 1.66, 2.40] remained significant. However, environmental factors analyzed further in the study were not significantly associated with the prevalence of diarrhea in children in underdeveloped regions of Indonesia.

**Table 3:** Multivariate logistic regression analysis of study variables among children under five years of age in underdeveloped regions of Indonesia (2018)

**Variables**	**OR (95% CI)**	***P *** **value**
Age 12-23 months	1.73 (1.48, 2.02)	0.001
Age 24-35 months	1.31 (1.11, 1.53)	0.001
Living in rural area	1.19 (0.95, 1.48)	0.132
Aware of any nearby hospital	0.74 (0.63, 0.86)	0.001
Diagnosed with ARI in the past month	1.99 (1.66, 2.40)	0.001
Diagnosed with pneumonia in the past year	1.39 (0.95, 2.04)	0.095
Household waste disposed of at random	1.11 (0.97, 1.28)	0.138
Child’s stool disposed improperly	1.11 (0.96, 1.28)	0.152
Drinking water from surface water	0.93 (0.70, 1.25)	0.645
Clean water source from surface water	1.25 (0.98, 1.59)	0.073
Constant	0.09	0.001

## Discussion

 This study assessed factors influencing diarrhea in children under five years of age in underdeveloped regions of Indonesia. The risk of diarrhea decreased with increasing age of the child. Toddlers aged 12-23 months had the highest risk of diarrhea compared to other age groups. This finding was consistent with a study conducted by Feti, which showed that children aged 6-23 months had a higher risk of suffering from diarrhea compared to older children. This increased risk was attributed to the reduced immunity obtained from the mother, the child’s growth and development, and the start of weaning at this age.^15^ Children under 5 years old are particularly vulnerable to diarrhea due to immature immune systems and poor sanitation, which hinder their ability to combat infections caused by pathogens such as *rotavirus*, *E. coli*, and *Shigella.*^16^ Furthermore, children in underdeveloped regions at this age might also suffer from low nutritional status. Low nutritional status can reduce the body’s immune reaction, making it more susceptible to infections that cause diarrhea.^17^

 When comparing the living areas, children under five years of age living in rural areas were more likely to suffer from diarrhea than those living in urban areas. This disparity can be attributed to factors inherent in rural settings. Rural areas often have limited access to clean water and adequate sanitation facilities, leading to higher exposure to pathogens that cause diarrhea.^18^ Additionally, healthcare services in rural regions are frequently less accessible and of lower quality compared to urban areas, resulting in delayed or inadequate treatment for diarrheal diseases. Poor infrastructure in rural areas can also contribute to the contamination of water sources with human and animal waste.^19^

 We found that awareness of the location of health facilities plays a crucial role in reducing the risk of diarrhea in children under five years of age. Parents or caregivers who are aware of the location of health facilities can seek prompt medical attention when a child shows symptoms of diarrhea. Early treatment can prevent complications, dehydration, and severe outcomes, reducing overall morbidity and mortality.^20^ Families are more likely to seek medical help when they know where to go and what services are available, leading to earlier and more effective treatment.^21^ Besides, awareness of local health facilities contributes to community-level surveillance and early warning systems for outbreaks of diarrheal diseases.^22^

 Other infectious diseases, such as ARI and pneumonia, were associated with increased child diarrhea in these regions. Diarrhea and ARI were the comorbid diseases that accounted for the main burden of morbidity and mortality in children under five years of age.^23^ Diarrhea can lead to pneumonia in undernourished children because of compromised immune systems.^24^ Undernourished children are also more likely to experience diarrhea, creating a vicious cycle. Children’s growth is hampered by pneumonia and diarrhea, with underlying malnutrition as a significant risk factor for both illnesses.^25^

 In this study, environmental factors such as drinking water sources and clean water sources from surface waters, as well as indiscriminate household waste disposal were significantly associated with an increased prevalence of diarrhea among children under five years of age. This study indicates that children consuming water from these sources are at higher risk of developing diarrhea, likely due to microbiological contamination and pollution. Surface water sources are often vulnerable to pathogen contamination, especially in areas with poor sanitation.^26^ Additionally, indiscriminate household waste disposal can pollute the surrounding environment, including water sources, thereby increasing the risk of diarrhea transmission among children. These findings underscore the importance of improving drinking water quality and implementing effective waste management practices such as preventive measures to reduce the prevalence of diarrhea in young children.^18^ However, these factors were not significant after multivariate logistic regression analysis. Certain aspects of these findings diverge from previous studies, while others align with them. For instance, the consumption of surface water has been linked to an increased risk of diarrhea among children in host communities in Ethiopia.^27^ This discrepancy may be attributed to the common practice of boiling water in Indonesian households, which reduces the risk of diarrhea.^28^ In line with the study from Ghana, a positive correlation has been observed between improper disposal of child feces and diarrhea in children under five years of age.^29^ However, household waste disposal showed no association with the prevalence of diarrhea, which is consistent with the findings of the study conducted in coastal communities in Ghana.^30^ This study suggested that non-environmental factors may play a more critical role in developing diarrhea in these regions, underscoring the complexity of public health issues in underdeveloped regions.

## Limitations

 This study included a comprehensive analysis of the factors that influence diarrhea in children under five years of age in underdeveloped regions of Indonesia, which is a significant public health problem. Using data from the 2018 National Basic Health Research, this study examined 9243 children from 60 regions to ensure a large and representative sample. These insights are important for developing targeted interventions to reduce the prevalence of diarrhea and contribute valuable knowledge to public health efforts in Indonesia. However, this study has limitations due to recall bias from participants concerning their medical history, as well as missing data on several variables that may affect the prevalence of diarrhea in underdeveloped regions of Indonesia, such as children’s nutritional status, hygiene and sanitation practices, and maternal or caregiver behaviors. Therefore, the results related to these variables cannot be explained in detail.

**Highlights**

- Toddlers are the most vulnerable group to suffer from diarrhea in underdeveloped regions of Indonesia.
- Children under the age of five in these regions who suffered from other infectious diseases, such as pneumonia and acute respiratory infections (ARIs), were more likely to suffer from diarrhea.
- Non-environmental factors play a significant role in the development of diarrhea among children under the age of five in these regions.

## Conclusion

 This study highlights that the risk of diarrhea decreases with the child’s age, with toddlers aged 12-23 months being at the highest risk due to factors such as reduced maternal immunity, growth, and weaning. Children under five are particularly vulnerable to diarrhea due to immature immune systems and poor sanitation. Rural areas show a higher prevalence of diarrhea than urban areas, which is attributed to limited access to clean water, sanitation, and healthcare. Awareness of nearby health facilities is crucial for reducing diarrhea risk by enabling timely medical intervention. Children with a history of respiratory infections are more susceptible to diarrhea, exacerbating their vulnerability. Environmental factors like contaminated surface water and improper waste disposal increase the prevalence of diarrhea, underscoring the need for improved water quality and waste management. The results of multivariate analysis conducted in this study confirm that the child’s age, history of ARI, and household awareness of nearby hospitals are critical factors influencing diarrhea in underdeveloped regions of Indonesia, while environmental factors play a less significant role compared to some other contexts. These insights are essential for developing targeted interventions to reduce the prevalence of diarrhea and improve children’s health in these areas.

## Acknowledgments

 The authors would like to thank the Ministry of Health of Indonesia for giving access to the data used in this study.

## Competing Interests

 The authors declare that they have no conflict of interests.

## Ethical Approval

 The 2018 RISKESDAS Protocol obtained Ethical Clearance from the National Ethics Committee of the Institute for Health Research and Development, Ministry of Health of the Republic of Indonesia, under approval number LB.02.01/2/KE.024/2018.

## Funding

 The authors did not receive any financial assistance.
